# Uncovering the protein aggregation process through effect of G41D mutant SOD1 charge variation in ALS disease

**DOI:** 10.1038/s41598-025-16910-9

**Published:** 2025-08-27

**Authors:** Zainab Abdullah Waheed, Abasalt Hosseinzadeh Colagar, Bagher Seyedalipour, Payam Baziyar

**Affiliations:** 1 Department of Molecular and Cell Biology, Faculty of Basic Science, University of Mazandaran, Babolsar, Iran; 2 Department of Medical Laboratory Techniques, Institute of Medical Technology Al-Mansour, Middle Technical University, Baghdad, Iraq

**Keywords:** Protein aggregation, fALS, SOD1, β-strands (β4), Molecular dynamics (MD) simulations, Biochemistry, Biophysics, Computational biology and bioinformatics, Neurology, Energy science and technology, Nanoscience and technology

## Abstract

Neurodegenerative disorders are a group of hereditary and sporadic conditions that are characterized by progressive nervous system dysfunctions. Mutations in the gene encoding human superoxide dismutase 1 (hSOD1) were among the first to be proposed in line with the protein aggregation theory for ALS disease. This study aimed to characterize the (G41D) mutation/charge effects on the biochemical and biophysical properties of the SOD1 structure through computational and experimental methods. The computed average values of RMSD, RMSF, and Rg demonstrate that mutation results in a loss of conformational stability, increased flexibility, and greater compactness, all supporting the observed aggregation. The G41D mutant revealed distinct changes in β-sheet content compared to WT-SOD1 under amyloidogenic conditions, as confirmed by FTIR spectroscopy. Furthermore, the formation of amyloid/amorphous species was identified using ThT/ANS fluorescence and confirmed by TEM analysis. Mutations that alter the net negative charge of the SOD1 protein are crucial in misfolding and shortening the lag phase in SOD1 aggregation. Our results provide supporting evidence that these charge alterations, alongside amyloid-inducing agents at near-physiological pH, significantly contribute to the formation of amyloid-like species. Therefore, studying the G41D mutation may provide valuable insights into the mechanisms of fALS-associated aggregate formation, which holds promise for the development of highly effective inhibitors in reducing aggregates and therapeutic potential.

## Introduction

Fibril assembly starts with the spontaneous and intricate physicochemical process known as protein folding. Misfolded species oligomerize to lead to additional misfolding and development of fibrillar structures^1^. A number of illnesses in humans, including the neurological conditions that accompany amyotrophic lateral sclerosis (ALS), are linked to the creation of hazardous protein aggregates^2^. Ninety percent of cases of ALS are sporadic (sALS), a fatal neurological condition; the other 10% of cases are familial (fALS)^3^. Recent evidence suggests that mutation in human Cu–Zn superoxide dismutase 1 (hSOD1) gene is one of the common factors associated with sALS and fALS^4^. So far, more than 230 missense mutations have been associated with approximately 20% of fALS cases and 3% of SALS cases with mutations in the hSOD1 gene^5^. Wild-type hSOD1 is of interest due to its higher stability, reduced oxidative stress, major contribution of β-barrel architecture (compact hydrophobic packing), dimerization-related hydrophobic interactions, coordination of metal ions, and an intramolecular disulfide bridge^6^. The aforementioned parameters are greatly impacted by the mutation, which also destabilizes the structure of hSOD1 and enhances its tendency to aggregate. However, the shape of the fibrils produced varies across mutants, and these variations are ascribed to modifications in protein dynamics and local residue structure^7^. Despite the significant differences in the pathogenic functions of hSOD1-associated mutations, several characteristics are shared by all of them^8^. It is yet unknown, nevertheless, how certain mutations impact disease survival or cause hSOD1 aggregation. It is now shown that the pathogenesis is caused by hSOD1 aggregates’ enhanced harmful function rather than its loss of function^9^. Decreased metal ion concentration^10^, reduced intramolecular disulfide bonds, loss of post-translational modifications, reduced charge (in certain cases), and disruption of the surface hydrogen bonding network all appear to contribute to and expedite the development of amyloid-like aggregates of mutant hSOD1 associated with ALS^11–21^. Prior research has demonstrated that hSOD1 folding (or misfolding) and monomer aggregation into dimers or fibrils are intricate, multistep processes^22^. Since hSOD1 is a highly charged protein, the consequences of electrostatics on its stability can vary depending on its charge patterning and net charge^23^. Additionally, oxidative stress, pH, and ionic concentration affect how stable highly charged proteins fold^24^. Understanding the interactions between different SOD1 proteins is crucial since dimerization of SOD1 is the initial stage in the creation of the native tetramer or other misfolded aggregates. Comprehending the thermodynamic factors that control protein stability is essential for developing the principles of protein folding as well as for treating neurodegenerative disorders. In order to further investigate this matter, we looked into a heterozygous mutation in exon 2 of the SOD1 gene, c.125G > A (p. Gly41Asp). TheG41Dmutation was first described by Rosenin1993^25^. In 1994, Rainero reported the same mutation in an Italian family across 5 generations, including 8 patients (6 males and 2 females) and 5 carriers^26^. Later, in 1997, Cudkowicz screened 290 ALS families in the United States to identify SOD1 mutations. Seven participants from the same family were identified as G41D carriers^27^. The G41Dmutation was first identified in a Chinese family by Niu in 2015^28^. Diverse clinical phenotypes among family members and cases reported in the literature may suggest a complex genotype–phenotype relationship of this mutation. In 2019, Tang reported 3 cases of G41D mutation^29^. Additionally, Liu and colleagues reported 7 G41D-mutated individuals in 24 fALS cases using a screening technique^30^. He claimed that the p.G41D mutation was more frequent in Chinese patients with SOD1-related ALS than in the Caucasian ALS group, suggesting a possible genotypic-geographical association of hereditary ALS. In the meanwhile, the ALS illness has been linked to the G41D mutation, which is found in the β4-strand. Research has shown that this mutation might cause protein aggregation by raising the net negative charge. Because of its steric obstructive nature and substitution that occurs outside of the Ramachandran plot’s permitted areas, the G41D mutant seems to be extremely destabilizing^31^. It makes sense to believe that the local networks of salt-bridge and hydrogen interactions in the solution and gas phase structures would change if a negative charge is introduced at residue 41 by substituting Asp for Gly. The protonated states of His-residues (His43 and/or His46) may have altered as a result of the G41D substitution, according to the findings of earlier research using Cu, Zn-SOD1, and G41D SOD1 Zn-bound^32^. Therefore, to understand the mechanism of change in the net charge of the protein in the mutant position, we investigated the aggregation tendency of residue 41 of the beta-4 strand as a function of increasing mutant charge. The precise specifics of how crowding and mutations modify the folding and aggregation processes as a whole are still unknown, despite these substantial experimental and theoretical investigations. Comprehending the thermodynamic factors that control protein stability is crucial not only for defining principles of protein folding in live cells but also for treating neurodegenerative disease treatments. Therefore, we used a combination of molecular dynamics (MD) simulations and experimental investigations to adequately characterize the effect of the G41D mutant on the biochemical and structural properties of SOD1.

## Materials and methods

### Computational methods

#### Investigation of structural changes and activity stability using bioinformatics methods

Numerous computational methods may be used to anticipate how amino acid substitution will affect the structure and function of proteins. The activity stability of the mutant protein was predicted using the Predict-SNP service^33^. Using the ΔΔG computations of the i-Stable, I-mutant2, and DUET servers, the impact of mutation on the stability and structure of hSOD1 was assessed. The free energy change (ΔΔG), as reported by these computation services, is positive for stabilizing mutations and negative for destabilizing mutations^34^.

#### Molecular dynamics (MD) simulation

The initial structure dimer of WT-SOD1 (chains A and B) for the MD simulations was obtained from PDB ID: 2V0A with a resolution of 1.15 Å. In this work, first, the desired PDB file was retrieved with the code 2V0A. We introduced mutations at specific sites within the structure and minimized energy using Molegro virtual docker. Moreover, the GROMACS 2020.1 software package and the CHARMM36 force field were utilized to do the MD-based simulations of WT and G41D mutant^35^. Thus, these simulations may aid in determining the destabilization and stabilization of the current processes as well as the eventual consequences of mutagenesis on the structure of proteins. Simple point charge (SPC) water model for solvation was used to create a topology file, the system was neutralized by adding the proper numbers of Na^+^ and Cl^˗^ ions, and the models were placed inside a dodecahedron box to do MD simulation. Cu and Zn metal ions settled into their corresponding binding sites. The steepest descent algorithm was used to minimize the system’s energy consumption^36^. Using the Parrinello–Rahman barostat with a 2 ps time coupling constant and the v-rescale thermostat with a 1 ps coupling constant, the temperature and pressure were maintained at 1.0 bar and 310 K, respectively. The systems were then exposed to 100 ps of NPT at 1 bar after being permitted to equilibrate under NVT for up to 100 ps at 310 K with 1000 kJ/mol restraint forces. A leap-frog method^37^ with a time step of 2 fs was used to integrate Newton’s equation into the MD simulation, and data was gathered every 10 ps. LINCS restrictions were used to keep the covalent bonds in the protein at consistent bond lengths^38^. Electrostatic interactions were calculated using the Particle Mesh Ewald (PME) method^39^. Ultimately, MD simulations were run for 200 ns at 310 K and pH 7.4 for the WT-SOD1 and mutant, in which every atom was free to move about, previously reported in other proteins^40–44^.

### Experimental section

#### Materials

SDS-PAGE chemicals and 1-Anilinonaphthalene-8-sulfonate (ANS) were obtained from Merck. Protein ladder, boric acid, uric acid, Tris/HCl, NaCl, Phenylmethylsulfonyl fluoride (PMSF), Dithiothreitol (DTT), Congo Red (CR), Thioflavin T (ThT), sodium acetate, isopropyl-b-D-thiogalactopyranoside (IPTG), Kanamycin, yeast extract, tryptone and agarose obtained from Bio Basic. 10–250 kDa Wide Range Blue-Red-Green Three-Color Protein Ladder, Prestained (Bio Basic-Canada). Nickel Nitrilotriacetic Acid (NTA) Agarose Resin affinity chromatography column [Agarose Bead Technologies (ABT)].

#### Site-directed mutagenesis Expression, purification and enzyme activity of WT-SOD1 and G41D mutant

The recombinant pET-28a (+) vector containing the hSOD1 gene (pET28a-SOD1) was used as the template as previously described^11^. The Quik-Change PCR site-directed mutagenesis was carried out for constructing single-point mutation G41D. A point mutation is created by designing two mutagenesis primers for G41D: forward primer (5ʹ TAAAGGACTGACTGAA**GAC**CTGCATGGATTCC 3ʹ) with mutation site (highlight), and reverse primer (3ʹ CGTAATTTCCTGACTGACTT**CTG**GACGTACCT 5ʹ) with mutation site (highlight). The product of PCR was digested with *Dpn I* to destroy methylated parental plasmid DNA. The pET-28a (+)-SOD1 was transformed into *E. coli* BL21 (DE3) cells by the heat shock (calcium chloride) procedure. Next, 5 ml of LB medium containing kanamycin (50 µg/mL) with a fresh single recombinant colony was inoculated and grown at 37 °C overnight with 200 rpm. Then 100 ml of LB medium containing 50 μg/ml kanamycin was inoculated with 500 μl of bacterial culture and 1 mM glucose overnight at 37°C with vigorous shaking until it reached an OD600 of about 0.6–0.8. After inoculation in a 100 ml culture, cells were induced with 0.8 mM IPTG, 7 mM lactose, 200 μM CuSO4, 60 μM ZnSO_4_ for 18–22 h at 22 °C and 220 rpm. The Cells were harvested by centrifugation (5000 rpm, 4 °C, 25 min). The supernatant was carefully removed, and the cell pellet was resuspended in lysis buffer [50 mM NaH_2_PO_4_, 300 mM NaCl, 10 mM imidazole, and 1 mM PMSF (pH 7.8)] and incubated with 1 mg/mL lysozyme on ice for 30 min and then the suspension was lysed by sonication (8 cycles, 20 s on, 40 s off). Finally, the total lysates were centrifuged at 12,000 rpm for 25 min at 4 °C to remove cell debris and the supernatant was collected and purified using Ni–NTA agarose affinity chromatography. The supernatant was loaded onto the Ni–NTA column, which was pre-equilibrated with equilibration buffer (50 mM NaH_2_PO_4_, 300 mM NaCl, 10 mM imidazole, pH 8). In the next step, the column was washed with 10 ml washing buffer (50 mM NaH_2_PO_4_, 300 mM NaCl, 20 mM imidazole, pH 8). Finally, the elution of the recombinant protein was performed with 2 mL elution buffer (50 mM NaH_2_PO_4_, 300 mM NaCl, 250 mM imidazole, pH 8). Since *E. coli*, as a prokaryotic model organism, lacks a post-translational modification system^45^, and due to the lack of a copper chaperone for SOD1 as metallized proteins with uniform copper and zinc content^46^, we planned to metallize the proteins by prolonged dialysis against CuSO_4_ and ZnSO_4_ solutions. A serial dialysis process was performed for the purified protein at 4 °C for several days, including metal removal, metal charging, and unbound metal removal^47^: (1) 10 mM EDTA and 100 mM sodium acetate pH 3.8, (2) 100 mM NaCl and 100 mM sodium acetate pH 3.8 to remove EDTA bound to hSOD1, (3) 100 mM sodium acetate pH 5.5 to remove NaCl. (4) 100 mM sodium acetate with 200 μM ZnSO_4_, pH 5.5, and (5) 100 mM sodium acetate with 200 μM CuSO_4_, pH 5.5. (6) Purified SOD1 proteins were dialyzed against 20 mM phosphate buffer at pH 7.4 for 24 h to remove unbound metals. Using BSA protein at various concentrations as a standard curve, the Bradford technique was used to calculate the total protein concentration. On a 12.5% SDS-PAGE gel, purified SOD1 proteins were seen and stained with Coomassie Brilliant Blue. The capacity of SOD1 to prevent pyrogallol autoxidation was used to measure its activity^48^. Utilizing the BioTek Epoch microplate spectrophotometer, the measurement was carried out. The quantity needed to prevent pyrogallol autooxidation by 50% per minute was determined as one unit of SOD activity.

#### Fluorescence spectroscopy

Intrinsic fluorescence was employed to perform studies utilizing a Jasco Fluorescence Spectrofluorometer (FP-8300). After dialysis of the pure SOD1, 20 µg/ml SOD1 in phosphate buffer (20 mM, pH 7.4) was used to measure the intrinsic fluorescence in a final volume of 2 ml. The excitation was fixed at 295 nm and emission spectra were recorded between 300 and 450 nm. The excitation and emission slit widths were set at 5 and 10 nm, respectively. By adhering to the hydrophobic patches of proteins, ANS was utilized in extrinsic fluorescence studies to monitor conformational changes in proteins. 10 μM of pure enzyme and 30 μM of ANS were incubated under amyloid induction conditions (50 mM DTT, 50 mM Tris–HCL, 0.2 M KSCN, pH 7.4 and at 190 rpm) at 37 °C for 0–72 h. The excitation was fixed at 350 nm and emission spectra were recorded between 400 and 650 nm.

#### FTIR spectroscopic studies

The protein’s secondary structure was examined using KBr tablets and Fourier-transform infrared spectroscopy (FTIR) (BRUKER TENSOR 27, Germany). A 4 cm^−1^ resolution was used to modify the infrared spectrum between 400 and 4000 cm^−1^. For 72 h, protein samples containing 30 μM under amyloidogenic conditions were incubated at 37 °C. Software called OriginPro 2021 was used to process the raw spectra of the amide I region (1600–1700 cm^–1^). In the quantitative examination of protein secondary structures, curve fitting of the FTIR in the amide I region is frequently utilized. When studying the components of the Amide I band using the FTIR curve fitting approach, overlapping peaks within the Amide I region are often seen using second derivative spectra and Fourier Self-Deconvolution (FSD).

#### Characterization of hSOD1 aggregation by Congo red (CR)

Congo red (CR) dye is used to identify β-sheet-rich amyloid fibrils; this results in a redshift from 490 to 540 nm. Therefore, the CR binding approach was used to further identify SOD1 aggregates. Thus, samples were incubated under amyloid induction conditions for varying durations (0–72 h) at 190 rpm in order to generate the amyloid of SOD1 (30 μM, dimer). Following an extended period of incubation at 37 °C, aggregated SOD1 samples were collected and combined with CR (20 μM) in a final volume of 300 μL. Prior to spectral analysis, the reaction mixture was incubated for 10 min at 25 °C. Using BioTek Epoch microplate spectrophotometry, the absorption spectrum was scanned in the 400–650 nm range.

#### Thioflavin T (ThT) fluorescence assay

The ThT fluorescence assay was utilized to monitor amyloid aggregate formation under inductive conditions as aforementioned. SOD1 concentration was 30 μM and ThT with a concentration of 20 μM was added to the incubated samples at different time intervals. The amyloid aggregate formation was monitored with excitation at 444 nm and through the increase in ThT fluorescence at 460–515 nm using a BioTek Synergy H4 Microplate Reader. The experiments were repeated three times. According to what was previously reported^49^, kinetic parameters were calculated using the following formula:

1 $$F = \frac{Fmax}{{1 + \exp \{ - \left[ {\left( {t - tm} \right)/\tau } \right]}}$$

F and F_max_ indicate the fluorescence intensity at time t and the end time of ThT signal, respectively. *t*_*m*_ represents the formation time of half of the amyloid aggregates. The term (*k*_*app*_) represents the apparent rate constant, which is also given by 1/*τ* for the increase in the length of fibrils, and the formula: *t*_*D*_ = *t*_*m*_ − 2*τ* is used to calculate the delay time (*t*_*D*_).

#### Transmission electron microscopy (TEM)

For TEM imaging, SOD1 fibrillation samples that were collected once the fibrillation reaction achieved saturation were utilized. In summary, 5 μL of aggregated samples were extracted and adsorbed onto 300-mesh copper grids coated with carbon for 5 min at various intervals. After applying 2% uranyl acetate (weight/volume) for negative staining, the sample was allowed to air dry for 20 s. A microscope (Philips, EM 208S, Netherlands) equipped with a 100 kV accelerating voltage was used to examine the samples.

## Results and discussion

### The substitution mutation modifies the β-barrel stability and surface electrostatic potential of SOD1

**Fig. 1:**
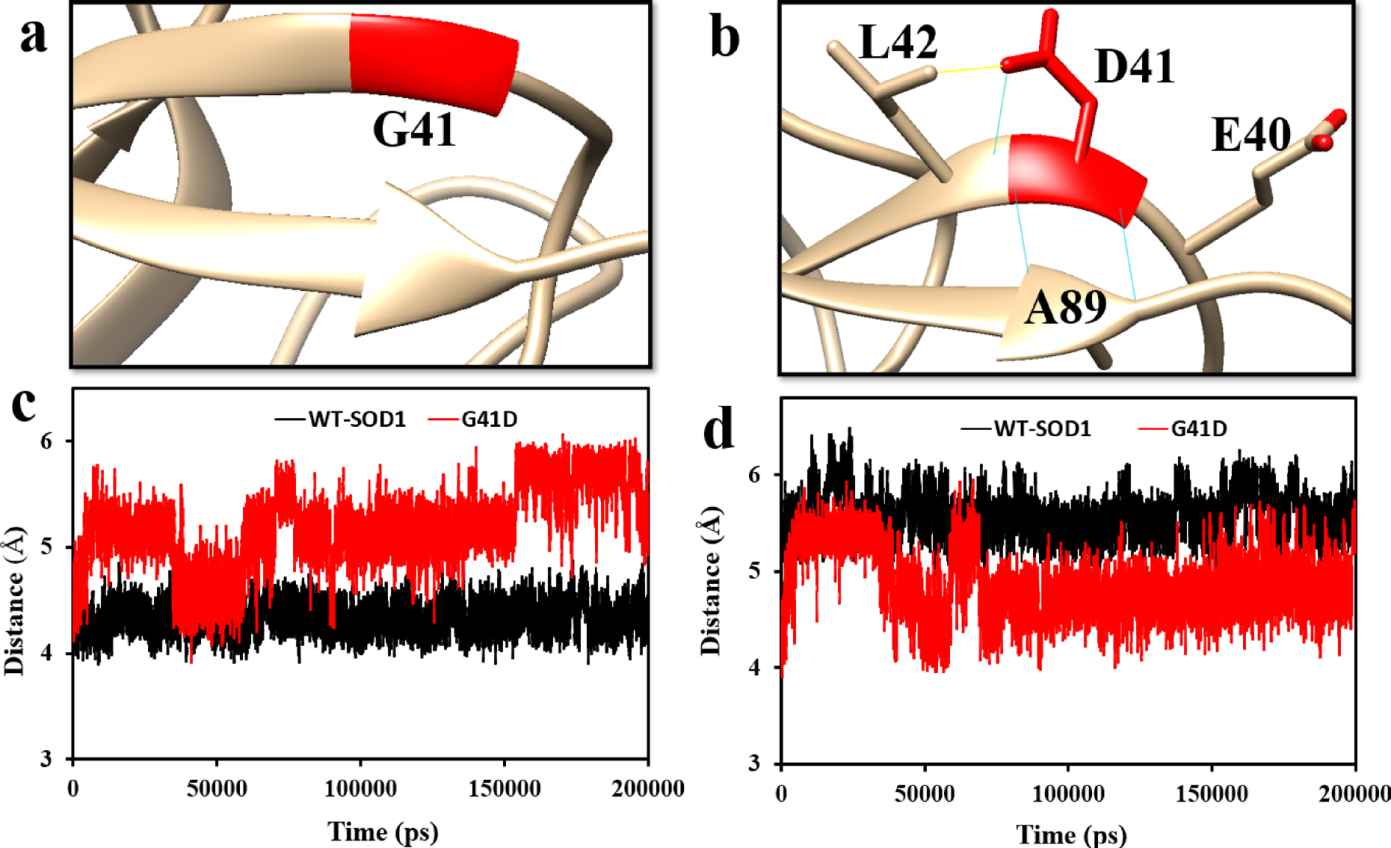
A general schematic of (**a**) WT-SOD1 and (**b**) location of G41D mutation. The blue and yellow lines represent the hydrogen bond and contact between the residues, respectively. The changes in the distance between the G41D mutant (red line) and the WT-SOD1 (black line) between (**c**) G41-E40 and (**d**) G41-L42 during the course of the simulation. The PDB entry 2V0A and the UCSF Chimera software were used to create the figure.

Protein misfolding has been observed as a result of β-4 strand mutations, which can interfere with the hydrophobic core of β-barrel packing. The stability of SOD1 β-barrel is altered by mutation at position G41. Increased net negative charge in protein by substituting Gly to Asp leads to an increase in aberrant contacts and improves the tendency to aggregate. Only Gly can benefit from the unique torsion angles (*ψ* and *ϕ*) that the WT-SOD1’sbackbonetakes at position 41. Therefore, mutations resulting in “less flexible” Asp residues cause backbone distortions that can influence the strength of the dimer interface as well as spread over large distances in the monomer structure. By destabilizing monomeric SOD1 and raising the likelihood of dimer-to-monomer dissociation, these actions lead to ALS. The most challenging case involves the mutation of Glycine at G41, which simultaneously causes large changes in chemical properties, volume, hydrophilicity, or both. Therefore, the G41D mutation was simulated using Chimera software and the results showed that the substitution of Gly instead of Asp at position 41 causes an aberrant increase in hydrogen bonding and intramolecular interactions (Fig. 1a and b).

The increased negative charge in the 41 mutant renders the protein’s net charge more negative than in the WT. Alterations in interprotein contacts involving residue 41 could elucidate the process of dimerization and further aggregation initiation. It has been proposed that the additional negative charge in the mutant reduces the interaction of residue 41 with residues 80–100 compared to the WT, while increasing its likelihood of interacting with residues 1–18 (β1) and 80–100 (β7, β8)^50^. Although it may be expected for a negative charge to interact with positively charged regions, it is crucial to observe that, despite β3 having a double positive charge, residue 41 in the mutant is unlikely to contact this region^51^. This might be because of steric effects between β6 and β3, indicating that electrostatics is not the only factor driving residue 41’s strong contact with residues 80–100. In SOD1 monomers, β7 and β8 are known to be the least stable and most flexible strands. Consequently, the G41D mutant’s displacement of β-strand partners by residue 41 may offer fresh perspectives on the process underlying the creation of fALS-associated aggregates^52^.

More specifically, we examined the average distance between residues 40–41 and 41–42 (WT-SOD1 and G41D mutant) during the simulation. In the WT-SOD1, the distance between G41-E40 was 4.33 Å, while in the G41D mutant, it increased to 5.2 Å (Fig. 1c). Subsequently, the average distance between G41-L42 in WT-SOD1 and G41D mutant was 5.58 and 4.74 Å, respectively (Fig. 1d). Due to changes in native contacts between the aforementioned residues, the stability of β-barrel may change and affect other interactions in the charging network. All things considered, our research indicates that mutations that neutralize or raise the protein’s net negative charge play a significant role in decreasing protein stability, which may cause misfolding and shorten the lag phase of the SOD1 aggregation process^53^.

#### SNP analysis

Single nucleotide polymorphisms, or SNPs, have been a crucial signal in genetic research and contribute considerably to the most frequent source of genetic variety in the human genome. Nonsynonymous DNA mutations can affect protein function by inhibiting structural modifications required for protein function, hence changing the amino acid sequence by increasing or decreasing protein stability^54^. Amino acids interact with their surroundings to form stable, three-dimensional structures in proteins. The protein-environment system in the minimal Gibbs free energy protein folding process (ΔG) consists of internal interaction energies, such as H-bonds, electrostatics, and hydrophobic interactions, along with the entropic contribution from hydrophobic effect and protein configuration^55^. Gibbs free energy of unfolding (ΔG) is a measure used to quantify the effect of non-synonymous mutations on protein stability. ΔΔG^*u*^ is a measure of the apparent free energy difference between mutant and WT proteins. Therefore, ΔΔG^*u*^ = ΔG^*u*^_*mutant*_ − ΔG^*u*^_*wild-type*_, which is the difference in unfolding free energy between mutant and wild-type proteins^56^. Then, using several methods (i-Stable, I-mutant2, and DUET) that take into consideration the variations in protein conformational stability brought about by replacement mutation, we projected observable stability changes and calculated the conformational instability linked to hSOD1. The results of the integrated servers’ calculations are shown in the Table 1. The discovery that for the mutation exhibited destabilizing effects on the WT-SOD1 structure helps to clarify how the G41D mutation impacts hSOD1. Previous research has demonstrated how missense mutations alter the stability of proteins^55^.

**Table 1:** Effect of mutation function on protein stability in silico analysis of SOD1 mutation.

Programs	G41D	Results
	∆∆G (Kcal/mol)	
I-mutant2.0 PDB	− 1.64	Decreases
AUTO-MUTE SVM	− 1.15	Decreases
AUTO-MUTE RF	− 1.86	Decreases
I-mutant2.0 SEQ	− 0.86	Decreases
i-Stable	− 1.34	Decreases
DUET	− 1.98	Destabilizing
Mcsm	− 1.7	Destabilizing
ENCoM	− 0.024	Destabilizing
SDM	− 3	Destabilizing
DynaMut	− 0.423	Destabilizing
DynaMut2	− 1.48	Destabilizing

Additionally, we evaluated how changes to amino acids impact the functionality of proteins using the computational methods of Predict-SNP. One way to predict if an option is harmful or neutral is to look at server and computational algorithm results. To link non-synonymous mutations to a variety of illnesses, we need a better understanding of the effect these changes have on protein stability. According to Table 2, the predicted confidence score for the G41D variation was 0.87, indicating that the Predict-SNP analysis results indicated negative effects^57^. To guarantee outstanding reliability, a number of criteria are applied. These results corroborate previous studies on other mutations (changes in protein stability)^21^.

**Table 2:** Predictions and confidence scores were obtained from six PREDICT-SNP server algorithms for the SOD1 mutation.

Programs	G41D	Predictions
	Confidence score	
Predict SNP	0.87	Deleterious
MAPP	0.91	Deleterious
Phd-SNP	0.82	Deleterious
PolyPhen-1	0.74	Deleterious
PolyPhen-2	0.59	Deleterious
SIFT	0.79	Deleterious
SNAP	0.81	Deleterious

#### MD simulation analysis

Amino acid changes in proteins can have detrimental effects on protein folding, stability, and metabolic processes. Molecular dynamics modeling was utilized to uncover the flexibility and structural variation of the SOD1 protein in order to examine the impact of mutation. Therefore, to study structural dynamic changes, MD simulations were run for 200 ns at 310 K on WT-SOD1 and G41D mutant.

**Fig. 2:**
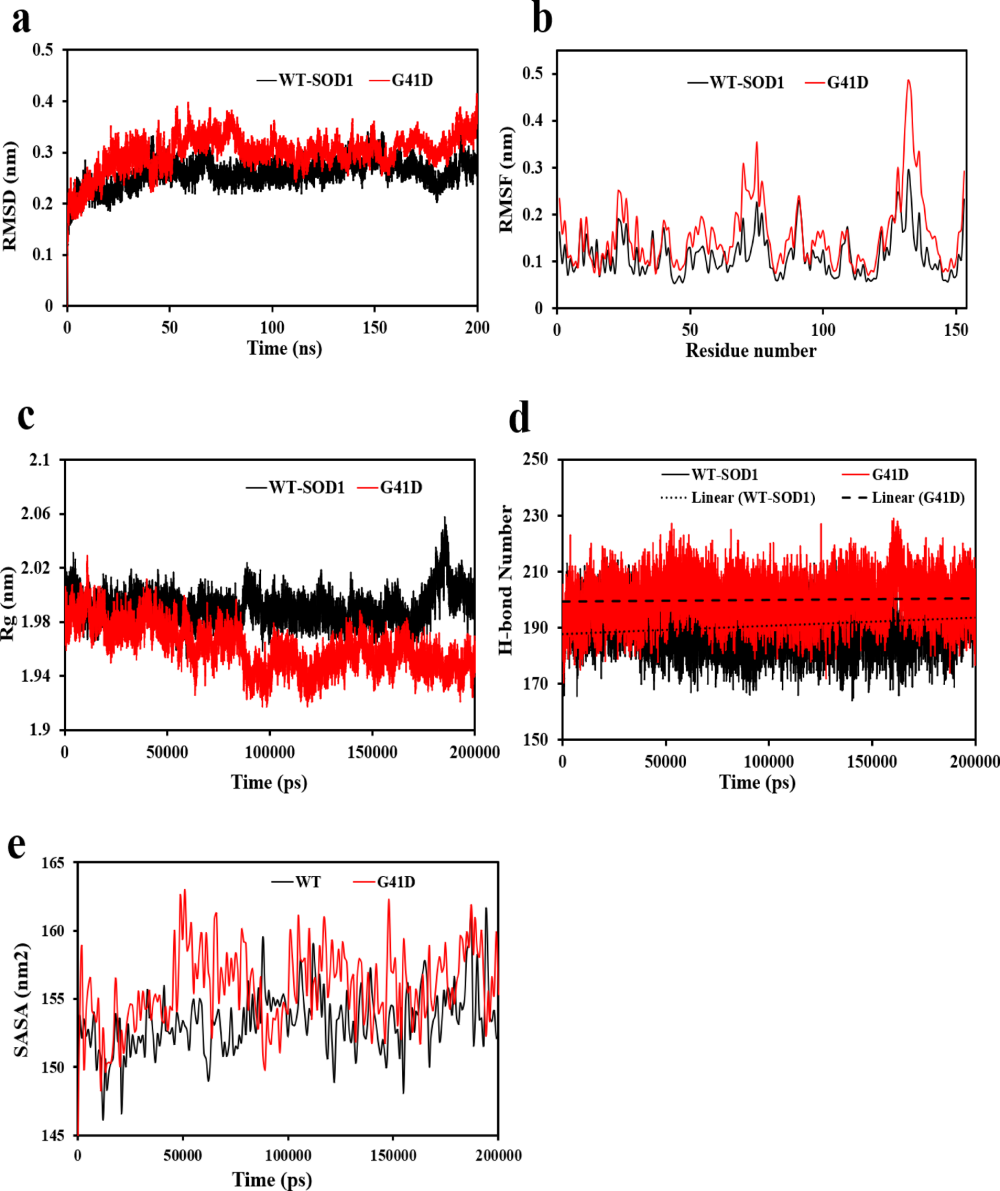
(**a**) Root-mean-square deviation (RMSD) for the WT-SOD1 and mutant backbone atoms is shown as a time function. (**b**) Root-mean-square fluctuation (RMSF) represents the degree of flexibility change WT-SOD1 and mutant protein. (**c**) Radius of gyration (Rg) of compaction changes for WT-SOD1 and mutant along the trajectory are shown. (**d**) Changes in hydrogen bonds for WT-SOD1 and the mutant along the trajectory are shown (**e**) Solvent-accessible surface area (SASA) of the WT-SOD1 and mutant during the MD trajectory are shown.

First, the difference between the final location of the Cα atoms in the protein backbone and its initial structural conformation was calculated using the root-mean-square deviation (RMSD). The structural conformational stability of a protein can be ascertained by changes that occur throughout its simulation^58^. The average RMSD values for WT-SOD1 and G41D mutant were 0.26 ± 0.014 and 0.31 ± 0.027 nm, respectively (Fig. 2a). These changes indicate that the substitution mutation has caused large structural fluctuations compared to the WT-SOD1. Under biochemical conditions, substituting Gly with a larger polar residue such as Asp may not only decrease the flexibility of the respective rings because the longer side chains of the substituted residues cause steric clashes with neighboring residues, but it can also alter the structural conformations of the protein^59^.

Next, we evaluated the residual flexibility of WT-SOD1 and G41D mutant proteins using root mean square fluctuation (RMSF). RMSF is a critical quantity that measures the deviation of a group of atoms from their average placement in a structural system using MD simulations^58^. During the simulation, the average flexibility values for the WT and mutant were 0.11 ± 0.04 and 0.15 ± 0.07 nm, respectively (Fig. 2b). Comparing the mutant to the WT-SOD1, more flexibility was seen in the residues comprising the functionally significant loops 49–83 (the zinc loop/loop IV) and 121–142 (the electrostatic loop/loop VII). This suggested severe deformation or disruption in these rings as a result of higher fluctuations over the simulation period. Loop IV’s position near the interface of the two monomers could significantly impact the stability of the dimeric structure of SOD1 due to observed fluctuations^60^. Thus, we deduced that the mutation changed the protein’s flexibility worldwide, which may be the primary cause of the protein’s loss of structural stability. As a consequence, the residual flexibility results agreed with the findings of the structural composition research, as previously reported^61^.

Similarly, a protein’s radius of gyration (Rg) tells us something about how relative compact the protein structure is, which has to do with how stable the protein is with the total of its intramolecular connections^62^. The mean Rg values for the WT and G41D mutant for the simulation were ~ 1.95 ± 0.011 and ~ 1.99 ± 0.016 nm, respectively (Fig. 2c). The findings demonstrated that the mutant structure was less stable than WT and had fewer intramolecular interactions. The computed average values of RMSD, RMSF, and Rg together demonstrate that mutation results in a loss of conformational stability as well as an increase in flexibility and compactness, all of which support the observed degree of aggregation^63^. H-bonds, another prevalent non-covalent interaction in proteins, are crucial for the structure and functionality of proteins^64^. H-bonds are the first bonds to react to structural abnormalities brought on by various protein mutations^65^. Thus, the SOD1 dimer underwent H-bonding. According to Fig. 2d, the mean H-bond values for the WT-SOD1 and G41D mutant were 189 ± 4 and 200 ± 6 H-bonds, respectively. The mutant has a substantial increase in the intermolecular H-bonds that form, which increases protein compaction. The outcomes align with the Rg parameter. The number of aberrant H-bonds is greatly increased as the protein’s surface becomes more negatively charged, strengthening the structure’s compaction and intramolecular contacts and eventually increasing the protein’s propensity to aggregate^66^.

Solvent-accessible surface area (SASA) is directly related to the surface area of a molecule that is accessible to solvent molecules, like water, and is a key factor in understanding molecular interactions. SASA is typically thought of as a method to assess how a protein interacts with solvents, which can then be utilized to determine the characteristics and function of the protein^61^. According to research that have recently been published, the structural composition of the protein has a significant influence on the surface properties, and even small changes in composition can alter the protein’s functional characteristics. All of the simulations’ SASA values (Fig. 2e) demonstrated consistent behavior. Over the course of the simulation, a rise in fluctuations was noted for the G41D mutant. This shift in dynamics results in structural instability, which in turn prompts the structure’s unfolding and eventual aggregation. The mean SASA values for the WT and mutant samples were 153 ± 2.31 and 155 ± 2.96 nm^2^, in that order. The variations in the exposure of buried hydrophobic core residues between the two systems were identified by means of the SASA study. Moreover, the higher SASA value revealed that the number of hydrophobic residues exposed to water allowed the protein to unfold, making the mutant easier to open than the WT. In contrast, the reduction in SASA value indicated protein folding, which coincides with an ejection of the hydrophobic side chains into the protein core, stabilizing the folded form. The SASA values, which show that the WT protein is in a folded state and the mutant protein is unfolding, provided a foundation for comprehending the events in the WT and mutant protein systems. The oscillations of the Rg and H-bonds shown in the simulations support these conclusions, which are consistent with previous studies^61,67^. Due to erroneous folding and relative compression brought on by this alteration, the dynamics of the structure become unstable, and aggregation increases.

Simultaneously, the Dictionary of Secondary Structure in Proteins (DSSP) tool was used to determine the secondary structure content of the WT-SOD1 and G41D mutant (Table 3). It should be highlighted that these secondary structures are highly significant since the tendency to alter the structure is a key factor in the aggregation process of proteins implicated in neurodegenerative diseases. The zinc and electrostatic loop residues of the WT and mutant exhibit the highest frequency of coil generation. Comparative analysis revealed differences in the tendency for coil formation between WT and the mutant. Additionally, the mutant exhibited a notable rise in the β-sheet content and a decrease in α-helix content compared to WT-SOD. Loops IV and VII of SOD1 include the α -helix, which is thought to be a functionally significant loop. The extension of loops IV and VII was brought on by a decrease in the α-helical content, which destabilized the protein and made it more prone to aggregation^68^.

**Table 3:** Alterations in secondary structure content of WT-SOD1 and G41D mutant.

System	Secondary structure elements (%)					
	Coil	β-Sheet	β-Bridge	Bend	Turn	α-Helix
WT-SOD1	29	34	4	17	12	4
G41D	27	37	3	19	12	2

### Purification and enzymatic activity of SOD1

**Fig. 3:**
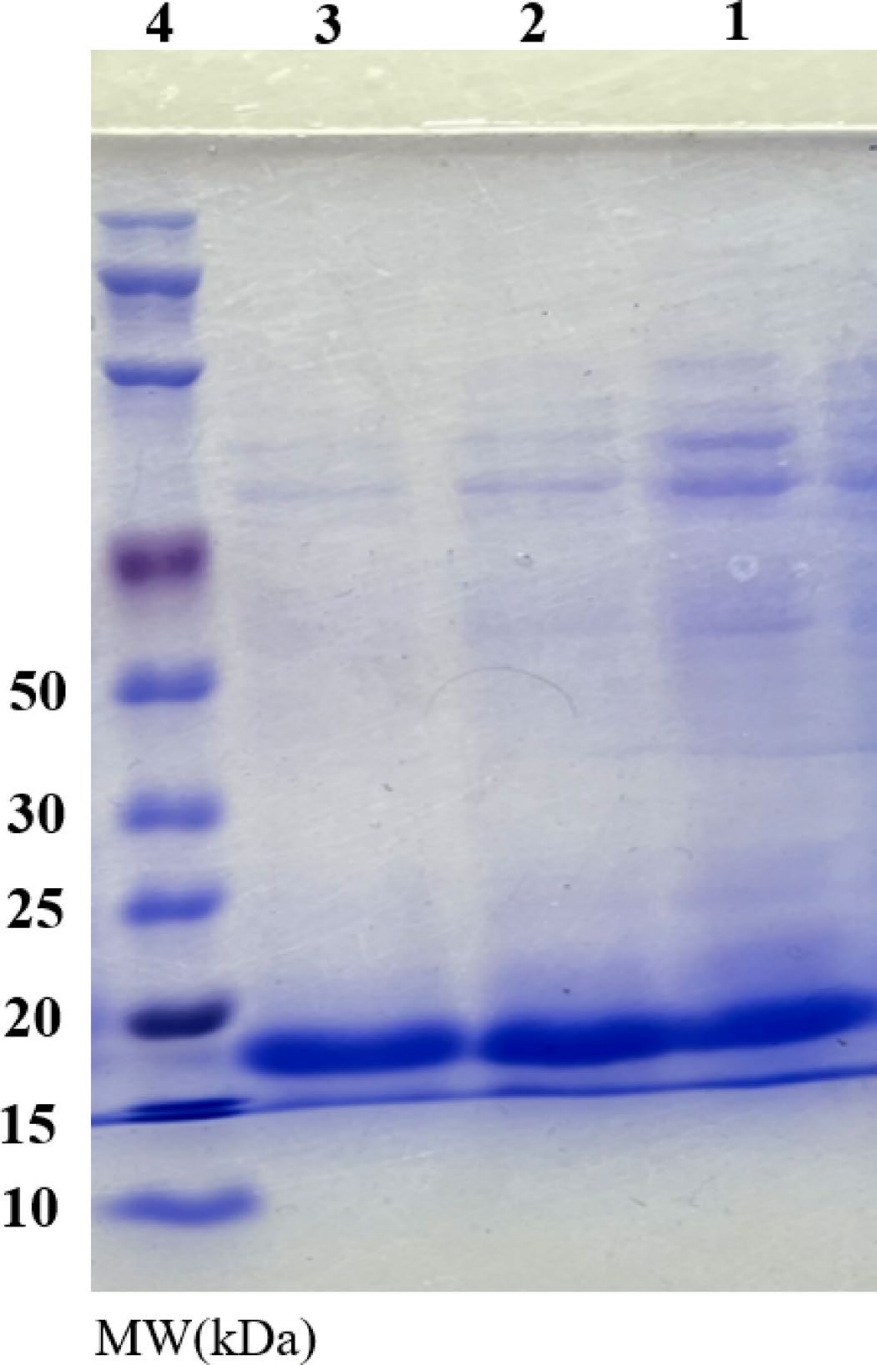
SDS-PAGE analysis (12.5% polyacrylamide gel) of purified enzyme by affinity chromatography under denaturing conditions and detected by Coomassie Brilliant Blue G-250. Lane 1and 2, show purified SOD1 G41D mutant using Ni–NTA affinity chromatography, which are elution buffers 1 and 2, respectively. Lane 3; show purified WT-SOD1. Lane 4; show molecular weight markers (10–250 kDa Wide Range Blue-Red Two-Color Protein Ladder).

Following Ni–NTA affinity chromatography purification of the SOD protein, its properties were determined on a 12.5% SDS-PAGE gel to validate the molecular weight and degree of protein expression. A single band with a molecular weight of around ⁓18 kDa was observed in the findings, and it represented the purity SOD1 protein that had been generated (Fig. 3).

Despite the remarkable stability of SOD1, structural instability brought on by point mutations encourages oligomerization and aggregation. Protein aggregation and misfolding are related to the loss of protein stability. A few mutations in the metal-binding loop associated with amyotrophic lateral sclerosis influence residues that decrease catalytic activity by coordinating Cu^2+^ and Zn^+^ ions. The enzyme activity of the WT-SOD1 and G41D mutant were (9508 ± 199) and (8471 ± 128) U/mg, respectively. Interestingly, mutations and changes in the protein net charge can result in an abnormal increase in H-bonds, which may cause a reduction in SOD1’s enzymatic activity, despite the fact that some H-bonds and intramolecular interactions are crucial for the folding and stability of SOD1^69^. All of the protein’s domains include disease-associated SOD1 mutations, and throughout time, efforts have been made to classify each variant’s impact based on its position and how it affects protein structure^70^. Two broad groupings have been proposed for SOD1 species based on their structural location and biophysical characteristics, including metal binding affinity and effect on SOD1 activity in vitro. Based on their SOD activity and metal-binding characteristics, these two sets of SOD1 proteins have been dubbed metal-binding region (MBR) and wild-type-like (WTL) fALS mutant SOD1 proteins^71^. Additionally, based on the RMSF data, loops IV and VII displayed greater alterations in the mutant during the simulation than did SOD1, suggesting a major malfunction or disorder in these loops. Alterations in flexibility occur in line with the loss of loop activity, which ultimately leads to the total elimination of SOD1’s catalytic activity. Additionally, the mutation induces a conformational shift that alters the orientation of the metal ligands involved, leading to a reduction in catalytic activity. Finally, the electrostatic loop generates an electrostatic field for the adsorption of superoxide anion radicals^60^. When combined, these findings imply that metal-binding plays a crucial role in SOD1 aggregation processes as well as being necessary for the enzyme’s enzymatic function. In particular, increased folding and aggregation in SOD1 are caused by abnormal metal binding in the mutant, suggesting the importance of metal binding in ALS pathogenesis^60^. Consequently, the structure and activity of the enzyme were significantly impacted by these localized or partial alterations.

### Fluorescence spectroscopy analysis

**Fig. 4:**
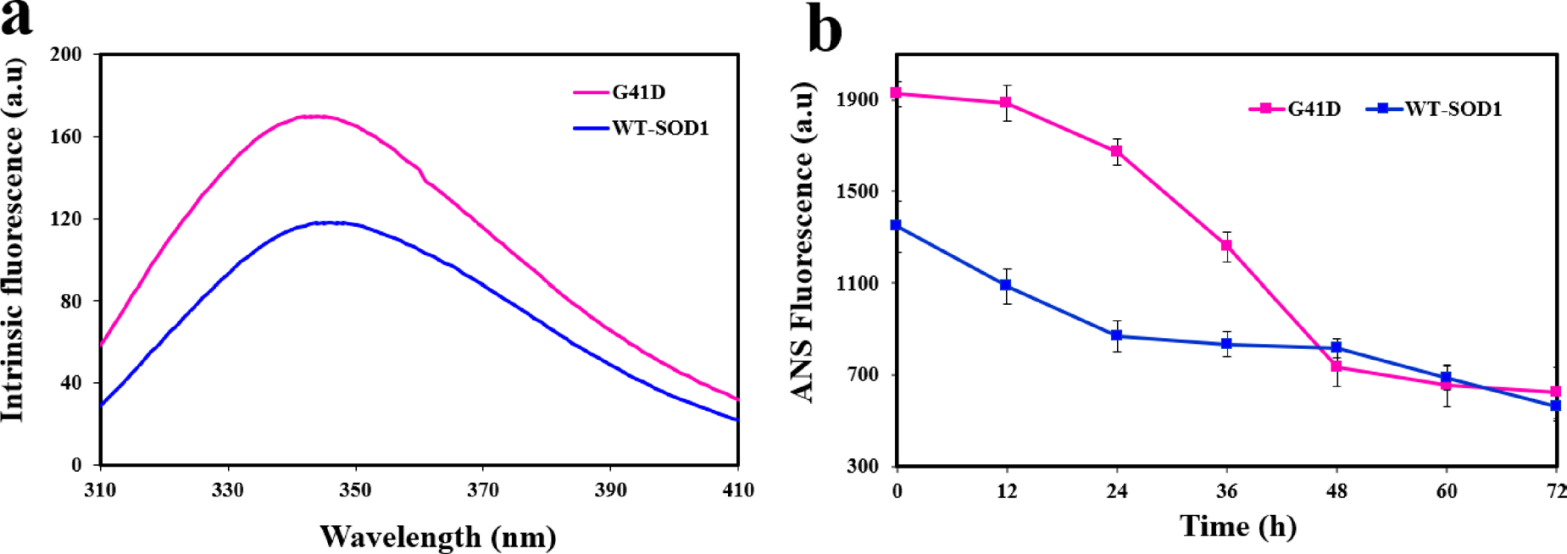
(**a**) Intrinsic fluorescence emission of WT-SOD1 and G41D mutant in 20 mM phosphate buffer. (**b**) Time-dependent ANS fluorescence emission of WT-hSOD1 and G41D mutant under amyloid-inducing conditions (50 mM DTT, 50 mM Tris–HCL, 0.2 M KSCN, pH 7.4 and at 190 rpm) at 37 °C for 0–72 h. The concentration of ANS was 30 μM and the molar ratio of protein to ANS was 1:30. Data were expressed as mean ± SD (n = 2).

Fluorescence spectroscopy has been demonstrated to be a useful tool for measuring the intrinsic fluorescence emission of tryptophan’s surrounding environment^72^. The impact of mutations on the conformational variations of WT-SOD1 was examined using a comparative structural analysis that included intrinsic fluorescence. The findings demonstrate that, as previously noted^73^, the mutations have varying degrees of impact on SOD1 conformation. The G41D mutant exhibits more intrinsic fluorescence emission intensity than the WT, suggesting that only tryptophan (W32) is positioned in a non-polar environment. Put differently, the Trp residue is situated in a more hydrophobic environment with less solvent exposure (Fig. 4a).

In order to examine whether the mutations under amyloidogenic settings alter the hydrophobic pockets of SOD1, we explored the exposure of hydrophobic patches during aggregation formation using the ANS probe. In order to identify compacted, partly folded stages of the protein population, ANS is employed as a charged hydrophobic fluorescent molecule^74^. Previous research has supported the hypothesis that a looser conformational state is responsible for the ANS probe’s binding to the intermediate surfaces of the mutant hSOD1^75^. Certain stages of SOD1 aggregation are probably aided by the hydrophobicity of the protein surface, and this parameter may vary in response to SOD1 mutation and metallization/demetallization^76^. Therefore, ANS fluorescence was used to assess the impact of mutation on SOD1’s tendency to form protein aggregates under circumstances that induce amyloid at 37 °C (Fig. 4b). When comparing the mutant to WT-SOD1, we found that the ANS fluorescence intensity was much higher, suggesting more hydrophobic patches in the proteins. It may be inferred that there are insufficient hydrophobic patches or contacts in the WT-SOD1 to contribute to the various stages of protein aggregation since the WT-SOD1 with lower ANS fluorescence has a lower aggregation potential than the mutant. This observation might imply that structural disruption, encompassing partial or total unfolding, is a necessary precondition for protein aggregation. However, WT-SOD1 has not undergone this process. However, as ANS is a sensitive protein conformation prob, the exposure of hydrophobic (micro) domains during SOD1 unfolding under aggregation circumstances is typically used to explain the spike in ANS fluorescence. In comparison to the wild type, ANS fluorescence intensity decreased with increasing incubation time, suggesting a rise in buried hydrophobic surfaces. Put differently, the expansion of aggregate species causes the hydrophobic surfaces that already exist to be covered, which inhibits ANS from attaching to hydrophobic pockets and lowers the amount of ANS fluorescence that is released. It is widely acknowledged that one of the primary mechanisms causing aggregation is hydrophobic effects. Under physiological circumstances, especially for mutant proteins, the reduction of intramolecular disulfide bonds is sufficient to promote aggregation in addition to the mutation made in the investigated SOD1, a dimeric protein where each monomer is linked to hydrophobic contacts. According to the ANS findings, protein aggregation is mostly caused by intermolecular hydrophobic interactions when DTT-mediated reduction of intramolecular S–S bonds results in extensive structural alterations^76^.

### Fourier transform infrared spectroscopy (FTIR)

**Fig. 5:**
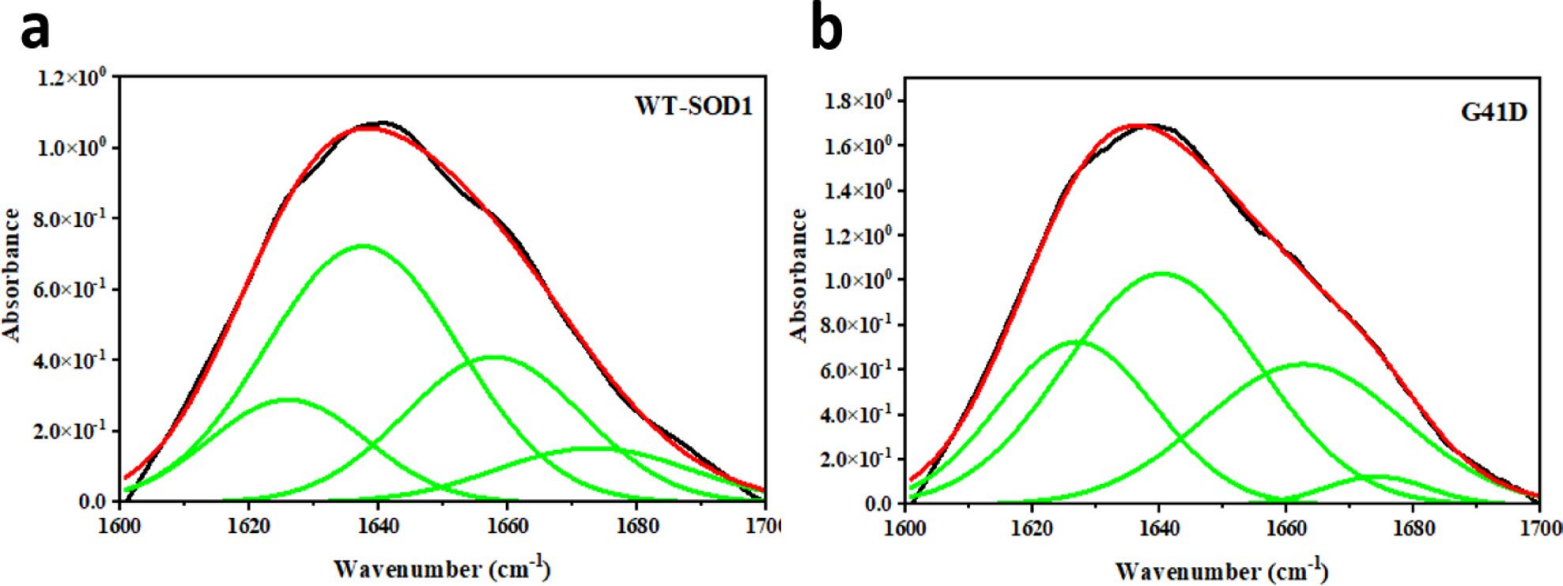
Results of a Fourier self-deconvolution (FSD) analysis on the amide I (1700–1600 cm ^−1^ region) for (**a**) WT-SOD1, (**b**) G41D mutant. Protein aggregates were prepared under amyloid-inducing conditions (50 mM DTT, 50 mM Tris–HCL, 0.2 M KSCN, pH 7.4 and at 190 rpm) at 37 °C for 0–72 h. The protein concentration was 30 μM.

FTIR spectroscopy is used widely in the study of protein secondary structure, misfolding, and aggregation formation^77^. As can be seen in Fig. 5, FTIR spectroscopy was utilized to show that secondary structure content had been created in both the mutant and WT-SOD1 after 48 h of incubation. Various β structure types and amyloidogenic conformers were identified using the FTIR spectra of the amide I regions. In proteins, stretching vibrations of the C=O peptide bond, which has the highest vibrational absorption, exhibit a noticeable and sensitive vibrational band in the 1700–1600 cm^–1^ region. A structure known for its unique IR signature, which consists of an increase in 1618 ± 10 cm^−1^ region, is responsible for the formation of amyloid-like aggregates^78,79^. It is possible to differentiate between the parallel and anti-parallel arrangements of β-strands in protein aggregates by examining the amide (I) regions. Only an increased component at 1630 to1636 cm^−1^ is seen in parallel β-sheets^80^. The assignment of bands around 1670–699 cm^−1^ to β-turns has been suggested^81^. Hence, the β-turns structure for WT-SOD1 and mutant was observed in the peak range of 1673 and 1674 cm^−1^, respectively. A component centered at approximately 1650 to1662 cm^−1^ is assigned to the α-helix, which is consistent with theoretical calculations and the observation of bands in the spectrum of α-helix proteins^82^. The α-helical structure for WT-SOD1 and mutant was observed at peak positions of 1657 and 1662 cm^−1^, respectively. As indicated by Table 4 data, WT-SOD1 showed a peak in the 1636 cm^−1^ range, which is specific to native β-sheets’ parallel architectures, while those from 1637 to 1644 cm indicate disordered structures (Fig. 5a). In the G41D mutant, variations in the proportion of β-sheet composition were noted. Furthermore, the G41D mutant’s findings showed a combination of intermolecular and parallel β-sheet structures, suggesting that β-sheet structure predominates in aggregation. For the G41D mutant, a peak was observed at 1641 cm^−1^, indicating the presence of disordered structures. (Fig. 5b). FTIR spectroscopy demonstrated that, in destabilizing circumstances, SOD1 displayed distinct alterations in the protein’s β-sheet structural contents as a result of the aggregation process. Therefore, SOD1’s structural rearrangement is indicated by alterations in secondary structure in SOD1 variations and the emergence of aggregation species. MD simulations demonstrate that these data are consistent with the β-sheet structural composition of SOD1 aggregates. Previous studies showed removing Cu and Zn ions, reducing disulfide bridges, and mutations associated with fALS in SOD all lead to the formation of aggregates with high β-sheet content, as confirmed by FTIR spectroscopy^18^.

**Table 4:** Assignment of amide I band components and percentages of secondary structure contents in WT-SOD1 and mutant.

System	Corresponding secondary structure	Observed negative peaks (cm^−1^)	Relative second structure (%)
WT-SOD1	β-Sheet	1636	45
	α-Helix	1657	28
	β-Turn	1673	12
	Intermolecular β-sheet (amyloid)	1625	15
G41D	β-Sheet	1641	45
	α-Helix	1662	25
	Intermolecular β-sheet (amyloid)	1626	25
	β-Turn	1674	5

Protein aggregates were prepared under amyloid-inducing conditions (50 mM DTT, 50 mM Tris–HCL, 0.2 M KSCN, pH 7.4 and at 190 rpm) at 37 °C for 0–72 h.

### Congo red (CR) binding assays

**Fig. 6:**
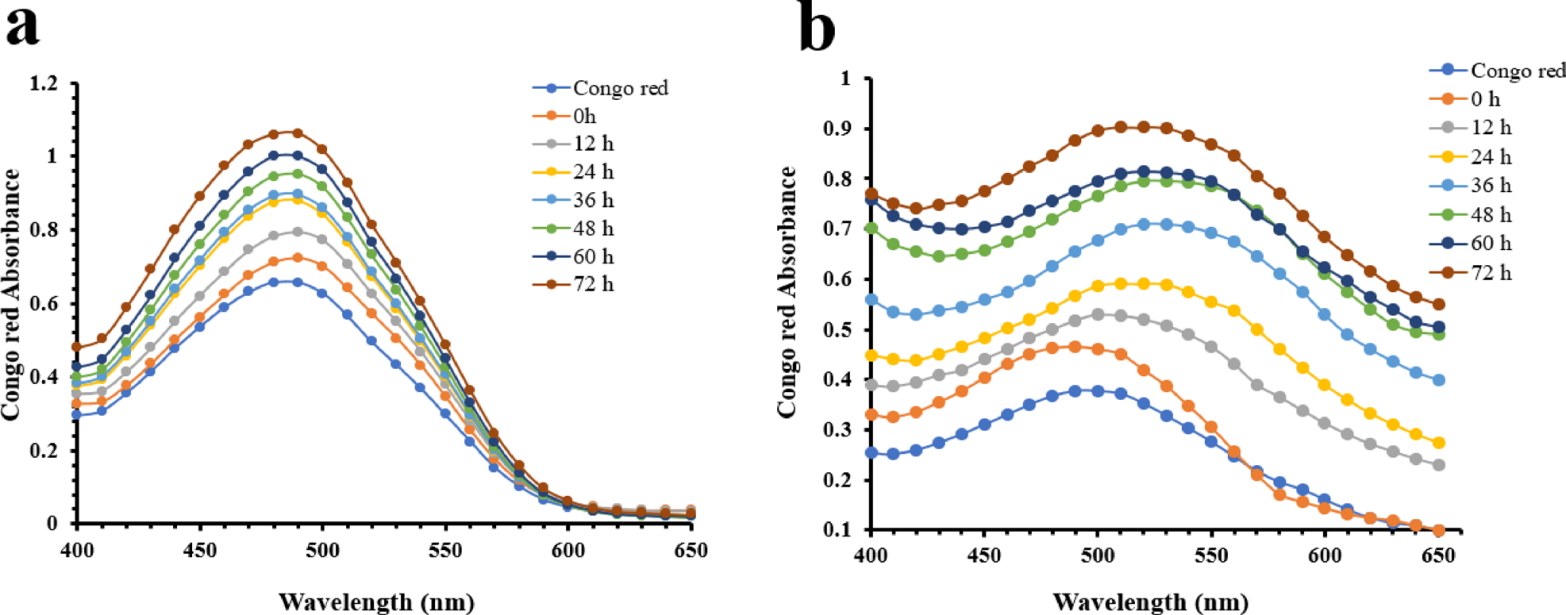
Congo red (CR) absorption spectra of (a) WT-SOD1, (**b**) G41D mutant. Protein aggregates under amyloid-inducing conditions Protein aggregates were prepared under amyloid-inducing conditions (50 mM DTT, 50 mM Tris–HCL, 0.2 M KSCN, pH 7.4 and at 190 rpm) at 37 °C for 0–72 h. The protein concentration and CR were 30 μM and 20 μM, respectively. The absorption spectra were recorded from 400 to 650 nm.

To investigate the formation of aggregates in the conditions of amyloid induction, the Congo red binding method was used. The binding of CR to the β-sheets increased the red shift of the absorption maximum at 490–520, indicating the characteristic of aggregate species. The binding of CR to the cross-β-sheet structure and amyloid fibrils is probably caused by the hydrophobic interactions^83^. Therefore, the formation of aggregate species under incubation and induction conditions of WT and mutant proteins with and without agitation (190 rpm) was studied for 0–72 h. The individual spectral features of the WT-SOD1 and G41D mutant are shown in (Fig. 6). However, we did not observe any changes in the absorption spectra of CR for the formation of aggregation conformers and prefibrillar species for the WT under amyloid-inducing conditions (Fig. 6a). As shown in Fig. 6b, increasing time-dependent incubation and the formation of aggregation conformers led to the greatest redshift observed in the mutant compared to the WT. These results indicate that increased beta-sheet content is associated with the highest levels of amyloid accumulation, as previous studies on SOD1 and other proteins have also confirmed^84,85^.

### ThT fluorescence assay

**Fig.7:**
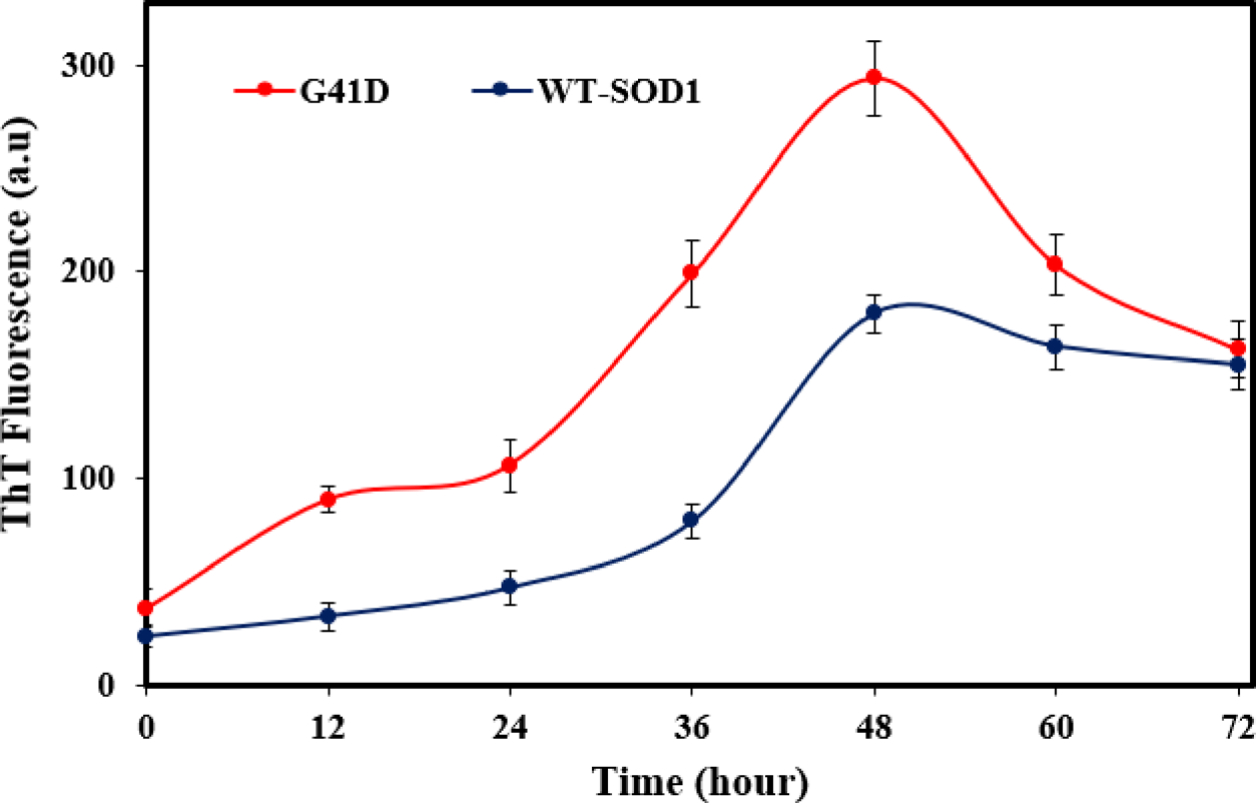
Effect of incubation conditions on the kinetics of amyloid aggregate formation of WT-SOD1 and G41D mutant using ThT fluorescence. For more information, please see “materials and methods” for amyloid-inducing conditions Protein aggregates were prepared under amyloid-inducing conditions (50 mM DTT, 50 mM Tris–HCL, 0.2 M KSCN, pH 7.4 and at 190 rpm) at 37 °C for 0–72 h. The protein concentration and ThT were 30 μM and 20 μM, respectively. Data were expressed as mean ± SD (n = 2).

Thioflavin T (ThT) fluorescence intensity end-point level is often used to infer mature fibril features. Recent structural models of amyloid fibrils suggest that ThT attaches to cross-β-sheet structures^86^. In this work, WT-SOD1 and its mutations were incubated for 0–72 h at 37 °C in the presence or absence of agitation (190 rpm) in the following conditions: 0.2 M KSCN, 50 mM DTT, and 50 mM Tris (pH 7.4). When KSCN was substituted for NaCl, no amyloid formed, indicating that KSCN’s actions are chaotropic in nature rather than merely a result of its ionic strength. ThT fluorescence and transmission electron microscopy (TEM) morphological studies demonstrated the formation of aggregated species inside the amyloid structure. A full set of kinetic parameters is provided in Table 5, and fluorescence time courses for WT-SOD1 and the mutant are displayed in Fig. 7.

**Table 5:** Kinetic parameters for amyloid aggregation formation from SOD1 and G41D mutant.

System	Lag time (h)	K_app_(h^−1^)
WT-SOD1	34 ± 2.82	0.25 ± 0.08
G41D	24 ± 2.41	0.016 ± 0.03

Protein aggregates were prepared under amyloid-inducing conditions (50 mM DTT, 50 mM Tris–HCL, 0.2 M KSCN, pH 7.4 and at 190 rpm) at 37 °C for 0–72 h. Data were expressed as mean ± SD (n = 2).

**Fig. 8:**
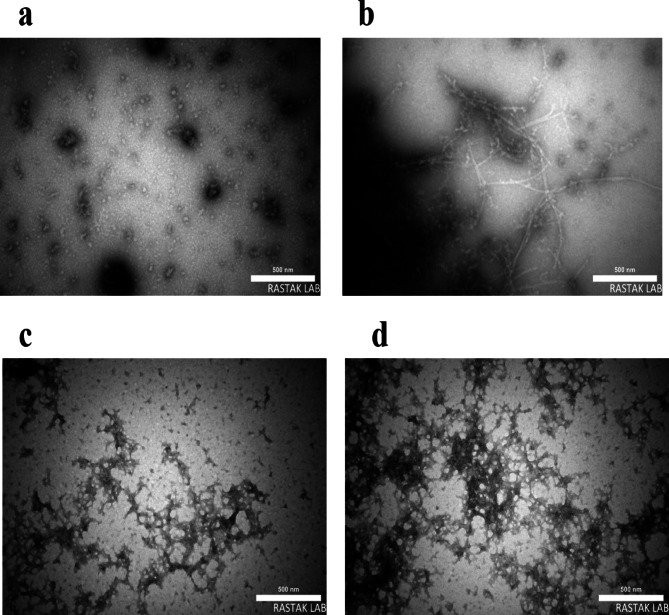
SOD1 samples (WT and G41D mutant) were imaged by transmission electron microscopy (TEM) under amyloid-inducing conditions Protein aggregates were prepared under amyloid-inducing conditions (50 mM DTT, 50 mM Tris–HCL, 0.2 M KSCN, pH 7.4 and at 190 rpm) at 37 °C for 0–72 h. (**a**) WT-SOD1 before incubation (**b**) WT-SDO1 48 h after incubation. (**c**) G41D mutant before incubation **d**) G41D mutant 48 h after incubation. (Scale bar = 500 nm).

Furthermore, our findings demonstrated that when exposed to large concentrations of DTT, both mutant and WT-SOD1 produce amyloid aggregates. There is a lag period in SOD1 fibrillation before ThT fluorescence rises. The WT-SOD1 and mutant showed differing lag phase lengths. Lag phase comparisons demonstrated that, in contrast to WT-SOD1, the mutant was more likely to accelerate fibrillation and decrease lag phases. The WT and mutant then displayed varying degrees of increases in thioflavin T fluorescence beyond this point. ThT fluorescence intensity differences arise from varying bound ThT molecule numbers and differing fluorescence quantum yields of fibrils^87^. The binding affinity and quantum yield of fibrils might vary depending on their morphologies, dynamics, and structures. As an illustration, fibril-to-fibril contacts, or “fibril matting”^88^, may result in decreased solvent accessibility or subsequent structural disturbances, both of which may lessen ThT binding^84,85^. This variation in the lag phase time is in line with results from earlier research that have been reported and examined in various contexts^20,73^. It should be highlighted, nonetheless, that a higher tendency for SOD1 aggregation and/or decreased latency may not invariably follow from mutations that cause fALS. The kinetics of amyloid fibril aggregation feature a lag phase followed by an increase in ThT fluorescence intensity, which is more pronounced in the mutant compared to the wild type. This might be because mat-like fibril aggregates generate particular modified or unreachable ThT binding sites. Conditions that are mutating or destabilizing might be the source of this variation in the kinetics of aggregation formation in SOD1. The plateau phase starts when the intensity of ThT fluorescence reaches its highest; nevertheless, it is short-lived since amyloid fibrils frequently form web-like network structures. According to previous research, dimer dissociation, metal ion loss from monomers, and weakening of the dimer interface are among the mechanisms that lead to SOD1 aggregation^89,90^. There are several aggregation mechanisms, some of which lead to amyloid aggregates and others to amorphous aggregates, according to earlier research. Furthermore, the findings demonstrate that hSOD1 aggregates amyloid in a range of circumstances, such as agitation, physiological pH, temperature, and ionic strength. We showed that, under amyloidogenic circumstances, both WT and mutant SOD1 have the capacity to generate amyloid structures. These findings imply that hydrophobic interactions account for at least some of the kinetic variations seen between protein aggregation. Therefore, hydrophobic interactions may accelerate fibrillization by raising SOD1 concentration and altering its conformation, promoting primary nucleation events. These results are in line with other research^11,18,20,73^ that highlighted the function of SOD1 fibrillation or oligomerization in the pathophysiology of ALS. TEM verified the existence of hSOD1 amyloid-like aggregates, as Fig. 8 illustrates.

### Transmission electron microscopy (TEM) of aggregates

Importantly, aggregates such as amorphous aggregates and amyloid fibrils are caused by misfolded proteins. Amorphous aggregates, which resemble amyloid fibrils and are formed by intermolecular interactions, are involved in the development of neurological disorders^91^. While the aggregation kinetics of amyloid fibrils and amorphous aggregates are similar, their morphology and stability are different, and they are typically hard to tell apart^92^. Despite amyloid fibrillation, thorough data about the kinetics of amorphous aggregation are scarce. Evaluation is therefore necessary in computational and experimental research. On the other hand, when proteins are denatured, their unfolded structure and exposed hydrophobic surfaces increase their ability to aggregate, which can result in amorphous aggregation. Finally, the presence of amyloid aggregates formed by incubating proteins under destabilizing conditions was confirmed by TEM images obtained with the ThT fluorescence technique. Specifically, the transmission electron microscopy images displayed several aggregate morphologies, including fibrillar, amorphous, and protofibrils. TEM images may be used to infer evidence of the aggregation mechanism at various stages of the process. TEM investigation was thus carried out for WT-SOD1 at time zero. In WT-SOD1, no morphological alterations linked to the development of intermediate amyloid fibrils were seen (Fig. 8a). Since a core structure is a fundamental component of SOD1 aggregates, we anticipate that mutation-dependent core structures will likewise have an impact on aggregate shape. Despite the fact that every mutant SOD1 aggregate examined here is fibrillar, there is a notable variation in fibril width between mutant SOD1. A thinner fibrillar filament did not develop in WT-SOD1 until 48 h of incubation at 37 °C (Fig. 8b). as demonstrated by earlier research^93^. The TEM morphology of the G41D mutant at time zero is seen in Fig. 8c. Following 48-h incubation period at 37 °C, samples were examined after the lag phase (the time interval that corresponds with the development of intermediate amyloid fibrils and the highest rise in fluorescence), frequently with fibrils or web-like networks present (Fig. 8d). Prolonged incubation resulted in the formation of large amyloid aggregates. As most reactions reach their elongation phase within 48 h, the mutant morphology transformed into amyloid aggregation, which manifested as fibrillar aggregates and rod-like structures^94^. There was a noticeable rise in the fibril development. The amyloid network and the extensive network of sporadic fibrils and amorphous filaments were seen when the majority of reactions reached the final plateau^4,95^. In addition, we saw significant morphological changes under different incubation conditions, such as prefibrillar species, networks resembling mesh, and long and short fibrils with or without branching fibrils. Examining the widths of these fibrils is challenging since it looks that tiny fibers are layered to build entwined and thicker fibrils with intricate forms. We have proposed that mutation-dependent core structures contribute to the range of aggregate morphologies that are produced, even if other structural and environmental variables will also influence the morphologies of SOD1 aggregates. The formation of amyloid-like aggregates is a prevalent characteristic under a variety of destabilizing situations, such as the decrease of disulfide bonds, the addition of DTT, and a rise in temperature. The findings also demonstrated that, in all unstable circumstances, the SOD had a strong tendency to form aggregates. All morphologies were found to be virtually amorphous but not fibrillar, despite a significant ThT signal, suggesting that the SOD1 structure was distorted into β-sheets, which is not characteristic of amyloids. These unexpected findings supported the images seen in earlier reports on SOD1 aggregates brought on by destabilizing circumstances^11,14,15,18,20^.

It can be helpful to create novel medicines, conduct more productive clinical trials, and provide genetic counseling when one understands the processes behind the beginning and course of a disease. Clinical trials for SOD1 antisense oligonucleotide therapies are in progress^96^. Therefore, classifying trial participants by SOD1 mutations and morphological features is essential for trial design and interpreting results. Overall, our data are consistent with the hypothesis that metal deficiency-induced misfolding may allow abnormal hydrophobic interactions of SOD1 with other cellular constituents or with itself, thereby exacerbating neurotoxicity^69^. To get a more profound understanding of both in vitro and in vivo processes, it is necessary to look at the function of physicochemical properties and mutations related to metal deficiencies. The contentious results (computational and experimental) about the accumulation processes and toxic effects of SOD1 variants linked to ALS serve as evidence for this. However, this finding helps investigators to treat the incurable form of ALS.

## Conclusions

Numerous unfolding factors, such as mutation, reduction of intra- and inter-disulfide bonds, changes in hydrophobicity, β-sheet tendency, solution composition, temperature, agitation, and pH, can lead to protein aggregation, misfolding, and various amyloid disorders. Therefore, understanding the SOD1 misfolding mechanism in neurons is crucial for ALS therapeutic development. The purpose of this study was to ascertain if different combinations and circumstances of incubation may make SOD1 variations more likely to aggregate or dissociate. The current findings highlighted the critical role of charged residues in the misfolding and aggregation of SOD1 variants. When compared to WT-SOD1, the mutant showed more pronounced alterations in flexibility, stability, protein hydrophobicity, and intramolecular interactions, according to MD data analysis. Given that the G41D mutation belongs to the WTL group, there was no appreciable variation in the activity of the mutant enzyme compared to the wild type. FTIR and DSSP were used to confirm the mutant’s increased inclination to generate beta sheets. Additionally, ThT/ANS fluorescence was used to identify the intermediate characteristics of amyloid/amorphous aggregates, and TEM imaging was used to validate them. Many characteristics of the aggregation of other disease-associated proteins are shared by the aggregate formation of SOD1 variations displayed here, such as the production of fibrillar and amorphous structures, the presence of a lag phase, and the rapid aggregate expansion caused by secondary nucleation. These findings provide insights into protein aggregation in disease, aiding future studies on mechanisms and therapeutic strategies against toxic aggregation linked to ALS.

## Data Availability

Data will be made available on request. If you request data, contact this Email: acolagar@yahoo.com or ahcolagar@umz.ac.ir.
